# Development and validation of three machine-learning models for predicting multiple organ failure in moderately severe and severe acute pancreatitis

**DOI:** 10.1186/s12876-019-1016-y

**Published:** 2019-07-04

**Authors:** Qiu Qiu, Yong-jian Nian, Yan Guo, Liang Tang, Nan Lu, Liang-zhi Wen, Bin Wang, Dong-feng Chen, Kai-jun Liu

**Affiliations:** 1 Department of Gastroenterology, Daping Hospital, Army Medical University (Third Military Medical University), Chongqing, 400042 China; 2 Department of Gastroenterology, People’s Hospital of Chongqing Hechuan, Chongqing, 401520 China; 3 Department of Medical Images, College of Biomedical Engineering and Imaging Medicine, Army Medical University (Third Military Medical University), Chongqing, 400038 China

**Keywords:** Multiple organ failure, Pancreatitis, Machine learning

## Abstract

**Background:**

Multiple organ failure (MOF) is a serious complication of moderately severe (MASP) and severe acute pancreatitis (SAP). This study aimed to develop and assess three machine-learning models to predict MOF.

**Methods:**

Patients with MSAP and SAP who were admitted from July 2014 to June 2017 were included. Firstly, parameters with significant differences between patients with MOF and without MOF were screened out by univariate analysis. Then, support vector machine (SVM), logistic regression analysis (LRA) and artificial neural networks (ANN) models were constructed based on these factors, and five-fold cross-validation was used to train each model.

**Results:**

A total of 263 patients were enrolled. Univariate analysis screened out sixteen parameters referring to blood volume, inflammatory, coagulation and renal function to construct machine-learning models. The predictive efficiency of the optimal combinations of features by SVM, LRA, and ANN was almost equal (AUC = 0.840, 0.832, and 0.834, respectively), as well as the Acute Physiology and Chronic Health Evaluation II score (AUC = 0.814, *P* > 0.05). The common important predictive factors were HCT, K-time, IL-6 and creatinine in three models.

**Conclusions:**

Three machine-learning models can be efficient prognostic tools for predicting MOF in MSAP and SAP. ANN is recommended, which only needs four common parameters.

**Electronic supplementary material:**

The online version of this article (10.1186/s12876-019-1016-y) contains supplementary material, which is available to authorized users.

## Background

Acute pancreatitis (AP) is a common and serious inflammatory disorder that may result in severe complications such as systemic inflammatory response syndrome (SIRS), organ failure, etc. The 2012 revised Atlanta classification stratified AP into mild acute pancreatitis (MAP), moderately severe acute pancreatitis (MSAP), and severe acute pancreatitis (SAP) based on the presence of persistent organ failure and complications [1]. If organ failure cannot be resolved within 48 h, SAP will develop. Single organ failure may persist to the late phase in AP, even affecting other organs [1]. The lung is the most commonly affected extrapancreatic organ in AP and this is frequently followed by acute kidney injury and cardiovascular system injury [2]. The main factor determining clinical outcome is the presence and duration of multiple organ failure (MOF) [1, 3], and the mortality of AP complicated by MOF is higher than 20% [4]. Since no specific drug is available to prevent AP developing into MOF, which is an extremely serious complication, it is pivotal to identify patients at high risk of MOF in an early phase, so intensive care and appropriate intervention can be provided to prevent disease progression.

Several single parameters such as C-reactive protein (CRP) and complex scores, including the Acute Physiology and Chronic Health Evaluation (APACHE) II score and Ranson score, are available to assess the severity of AP. However, their practical application for predicting the risk of MOF in an early phase is limited, since the CRP value can only indicate the inflammation state 72 h after the onset of symptoms; in addition, these scoring systems are cumbersome and require that some indexes are recorded dynamically [1]. At present, no single parameter or system is capable of predicting MOF in AP accurately. Therefore, it is essential to develop and validate a prognostic tool that can reliably predict MOF in the early phase.

MOF in SAP is thought to be a consequence of many factors, including uncontrolled systemic inflammation, microcirculation disturbance, coagulation dysfunction, and so on. SAP is often accompanied by substantial changes in the coagulation system [5], and coagulation-inflammation interactions occur in SAP [6]. Therefore, we speculated that inflammatory and coagulation markers could be helpful for predicting the risk of MOF. Moreover, blood urea nitrogen (BUN) is associated with mortality in AP [7] and creatinine is shown to be a marker of pancreatic necrosis [8], so they can probably predict MOF in AP as well.

It is well known that machine-learning techniques such as support vector machine (SVM), logistic regression analysis (LRA) and artificial neural networks (ANN) provide new methods for predicting clinical outcomes and complications at an individual level, and these have already been applied to clinical studies [9]. The aim of this study was to develop a computational tool for predicting the risk of MOF in MSAP and SAP from a larger set of parameters that include blood volume, inflammatory, coagulation and renal function markers, which have been shown to be different between patients with and without MOF. Five-fold cross-validation was used to test the predictive ability of SVM, LRA and ANN, and we compared the predictive efficiency of the three models and APACHE II score.

## Methods

### Patients and data collection

This retrospective cohort study was performed in three affiliated hospitals (Daping Hospital, Southwest Hospital and Xinqiao Hospital) of Army Medical University, Chongqing, China. Data of MSAP and SAP patients were collected from July 1st, 2014 to June 30th, 2017. Diagnostic criteria of MSAP and SAP followed the consensus revision of the Atlanta classification [1]. The patients with conditions such as pregnancy, pancreatic cancer, liver cirrhosis, coagulation system disease and incomplete laboratory examinations and those who were transferred after initial treatment or the time from onset to hospital admission exceeded 24 h were excluded from this study. According to the guideline of management of AP [10], all patients underwent standard medical treatment such as early aggressive hydration, antibiotics for infection, enteral nutrition, and so on. The Modified Marshall score was used to evaluate organ failure at 48 h after admission to determine whether they had delayed MOF including the pulmonary system, renal system and cardiovascular system failure. The study protocol was reviewed and approved by the Ethics Committee of Army Medical University and all methods were performed in accordance with the relevant guidelines and regulations. As a retrospective observational study and all subjects were anonymized, informed consent was not required.

Demographic and clinical features, including gender, age, body mass index (BMI), history of hypertension, diabetes and etiology (biliary, hypertriglyceridemia, alcoholic, et al) were recorded. Laboratory data, a total of twenty-three parameters obtained on admission, are shown in Additional file 1. Meanwhile, we calculated the admission APACHE II score.

### Statistics

Descriptive data were presented as median and interquartile ranges for skewed distribution variables or mean ± standard deviation for variables with a normal distribution. Categorical data were presented as proportions. The Pearson chi-square test was used to compare categorical variables and multiple rates. A *t*-test and the nonparametric Mann-Whitney test were used to compare normal and skewed distribution variables, respectively. Statistical analyses were performed using SPSS 23.0 software. Comparison for multiple ROC curves of three models and APACHE II score were performed using MedCalc software.

### Machine learning model

SVM, LRA and ANN were performed with Matlab 2014. The selected parameters entered into SVM, LRA and ANN were variables that had a significant difference (*P <* 0.05) in univariate analysis of the whole cohort. As output, a binary variable was used with one category representing some patients with MOF (1) and the other representing patients without MOF (0). All original values were used for LRA, while they were normalized, ranging from − 1 to 1, for SVM and ANN. A feature selection process was used to incrementally choose the most representative features and increase the relevance and reduce redundancy of prediction. In an attempt to prevent overfitting, given the limited training cohort available, and to maximize generalizability, we used five-fold cross-validation to train classifiers. The whole dataset was randomly divided into five roughly equally numbered, non-overlapping subsets, each called a fold. Then, four of the five folds were used as the training set, and the remaining one as the validation set. Using each of the five folds as validation set, the above process was repeated 10 times. We set two parameters in SVM with radial basis function (*C* = 1, gamma = 0.5). Finally, the final receiver operating characteristics (ROC) curve and averaged area under the curve (AUC) value of these three classifiers for the 10 trials were obtained to assess the classification algorithm.

With predicted the pseudo-probability from SVM, LRA and ANN, and obtained the cutoff value from ROC curve. Then, sensitivity (SEN), specificity (SPE), false positive rate (FPR), false negative rate (FNR), positive predictive value (PPV), negative predictive value (NPV) and accuracy were calculated from these three models. We compared these indexes of these models and APACHE II score.

## Results

### Baseline characteristics

Two hundred and 63 patients suffering from MSAP and SAP were enrolled in this study. The characteristics of the included patients with and without MOF were summarized in Table 1. The characteristics of the whole cohort of patients are shown in Additional file 2. Seventy-two (27.38%) patients suffered from MOF. Consistent with previous reports [11, 12], biliary tract disease (40.30%) was the most common cause of AP, and hypertriglyceridemia came second (34.98%). One hundred and 47 of all the patients were obese (BMI ≥ 25 kg/m2) [13].

**Table 1:** Characteristic of patients in group with MOF and without MOF

	No MOF (*n* = 191)	MOF (*n* = 72)	Statistic	*P* value
Male, no. (%)	123 (64.40%)	42 (58.33%)	*x*^2^ = 0.823	0.364
Median age, year	47.00 (39.00–59.00)	47.50 (39.00–58.75)	Z = − 0.266	0.791
History of hypertension, no. (%)	39 (20.42%)	19 (26.39%)	*x*^2^ = 1.084	0.298
History of diabetes, no. (%)	23 (12.04%)	8 (11.11%)	*x*^2^ = 0.044	0.835
Etiology, no. (%)			*x*^2^ = 2.968	0.397
Biliary	75 (39.27%)	31 (43.06%)		
Hypertriglyceridemia	66 (34.56%)	26 (36.11%)		
Alcoholic	21 (10.99%)	3 (4.17%)		
Other	29 (15.18%)	12 (16.66%)		
BMI, kg/m2	25.55 ± 3.89	25.82 ± 3.10	t = − 0.591	0.555
Obese (BMI ≥ 25 kg/m2), no. (%)	105 (54.97%)	42 (58.33%)	*x*^2^ = 0.239	0.625
Routine blood test				
WBC, × 109/L	13.47 (8.95–17.29)	12.97 (10.03–16.73)	Z = − 0.046	0.963
NEUT, %	86.00 (80.50–89.80)	85.2 (79.55–89.88)	Z = − 0.357	0.721
HCT, %	37.00 (30.50–43.60)	31.65 (25.05–43.73)	Z = −2.696	**0.007**
PLT, × 109/L	166.00 (124.00–227.00)	145.50 (84.50–247.75)	Z = − 1.733	0.083
MPV, fL	12.40 (10.90–13.90)	12.10 (10.93–13.80)	Z = −0.675	0.500
PDW, %	16.60 (12.80–20.07)	16.75 (15.00–18.30)	Z = −0.414	0.679
Coagulogram				
PT, seconds	12.70 (11.80–13.70)	14.35 (12.33–16.10)	Z = − 4.519	**< 0.001**
APTT, seconds	29.80 (27.10–33.00)	37.40 (30.93–47.00)	Z = − 5.243	**< 0.001**
TT, seconds	14.90 (13.80–16.90)	17.00 (15.33–21.35)	Z = − 4.748	**< 0.001**
FIB, g/L	4.60 (3.56–5.90)	3.73 (2.58–4.64)	Z = − 4.191	**< 0.001**
D-dimer, mg/L	2450.00 (1010.00–4983.00)	3219.00 (1371.88–5972.50)	Z = − 1.980	**0.048**
TEG				
R-time, minutes	5.60 (4.60–6.60)	6.35 (4.80–8.78)	Z = − 3.014	**0.003**
K-time, minutes	1.40 (1.10–1.80)	1.80 (1.30–2.90)	Z = − 4.316	**< 0.001**
α, degrees	70.10 (64.80–73.50)	64.80 (53.90–72.60)	Z = − 3.901	**< 0.001**
MA, mm	68.90 (63.50–73.50)	63.55 (54.85–70.68)	Z = − 3.609	**< 0.001**
Ly30, %	0 (0–0.30)	0 (0–0)	Z = − 1.655	0.098
CI	1.90 (0.40–2.80)	− 0.05(− 3.83–2.38)	Z = − 4.403	**< 0.001**
Inflammatory markers				
CRP, mg/L	122.30 (31.30–200.00)	175.65 (85.73–200.00)	Z = − 2.247	**0.025**
IL-6, pg/ml	33.00 (6.20–95.50,)	99.95 (44.80–293.90)	Z = −5.612	**< 0.001**
PCT, ng/ml	0.80 (0.23–1.84)	4.75 (0.60–19.44)	Z = − 5.591	**< 0.001**
Renal function				
BUN, mmol/L	5.20 (3.61–6.96)	8.05 (5.27–16.40)	Z = −5.334	**< 0.001**
Creatinine, μmol/L	64.20 (51.00–85.30)	119.35 (57.00–274.15)	Z = −4.793	**< 0.001**
Ca^2+^, mmol/L	1.98 (1.79–2.14)	1.96 (1.72–2.16)	Z = − 0.335	0.737
APACHE II score	9.00 (7.00–11.00)	14.00 (11.00–15.75)	Z = −7.879	**< 0.001**

Entries in boldface showed significant difference

Parameters including hematocrit (HCT), coagulogram, thrombelastogram (TEG), inflammatory markers, renal function and the APACHE II score differed significantly between patients with and without MOF (*P* < 0.05). Unexpectedly, no statistical differences were observed in the levels of white blood cell (WBC) count and calcium ion (Ca^2+^) between the two groups (*P* > 0.05). Meanwhile, no differences were observed in gender, age, history of hypertension and diabetes, etiology and BMI between the two groups (P > 0.05).

### SVM prediction

**Fig. 1:**
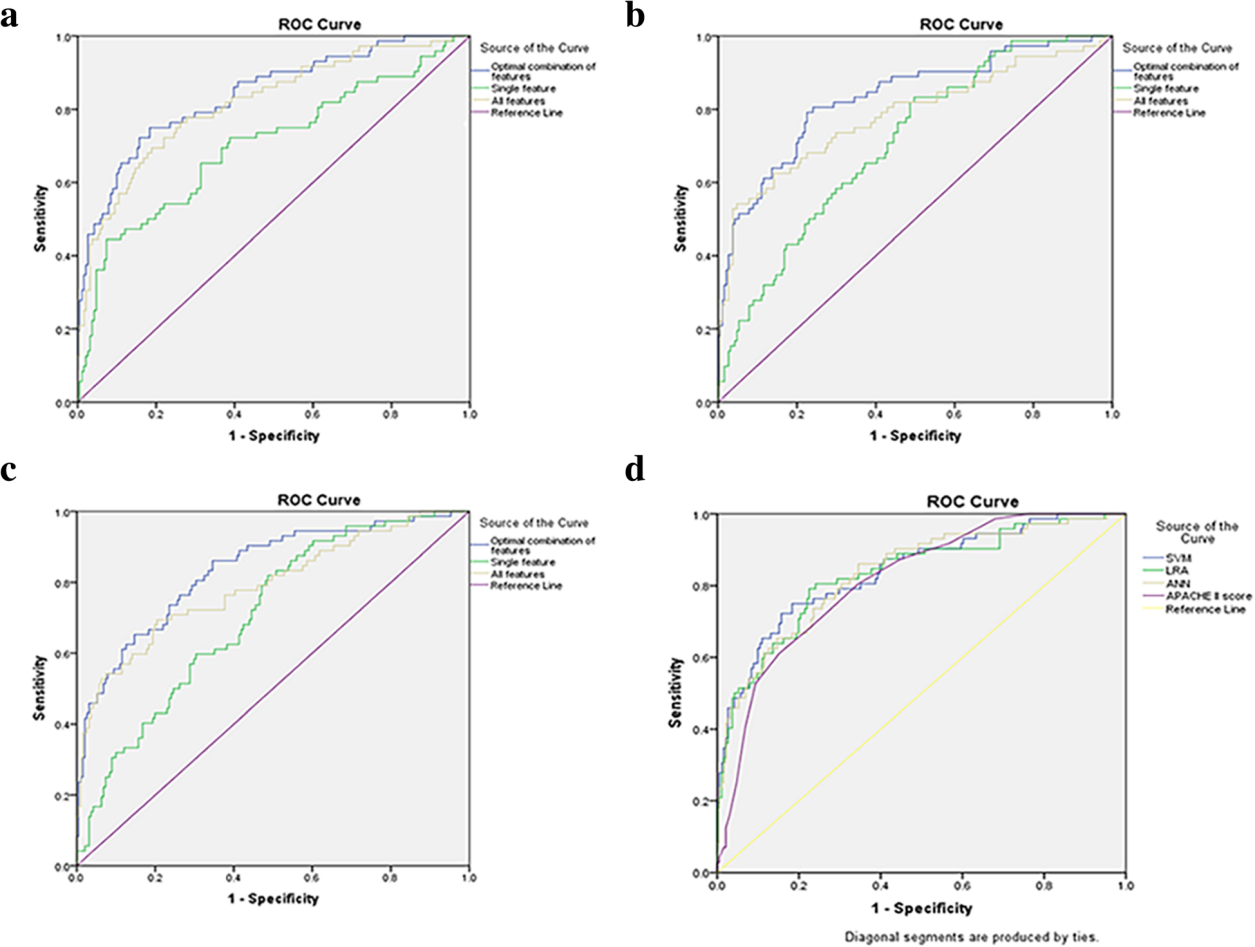
The ROC curves of different models. **a** The ROC curves of different combinations of features from SVM for predicting MOF in MSAP and SAP. AUC of the optimal combination = 0.840 (95% CI: 0.783–0.896); AUC of single feature (BUN) = 0.702 (95% CI: 0.625–0.778); AUC of all features = 0.816 (95% CI: 0.755–0.876). **b** The ROC curves of different combinations of features from LRA for predicting MOF in MSAP and SAP. AUC of the optimal combination = 0.832 (95% CI: 0.773–0.890); AUC of single feature (IL-6) = 0.709 (95% CI: 0.642–0.775); AUC of all features = 0.783 (95% CI: 0.714–0.853). **c** The ROC curves of different combinations of features from ANN for predicting MOF in MSAP and SAP. AUC of the optimal combination = 0.834 (95% CI: 0.777–0.890); AUC of single feature (IL-6) = 0.705 (95% CI: 0.639–0.772); AUC of all features = 0.789 (95% CI: 0.723–0.856). **d** The ROC curves of three models and the APACHE II score for predicting MOF in MSAP and SAP. AUC of SVM = 0.840 (95% CI: 0.783–0.896); AUC of LRA = 0.832 (95% CI: 0.773–0.890); AUC of ANN = 0.834 (95% CI: 0.777–0.890); AUC of APACHE II score = 0.814 (95% CI: 0.759–0.869)

Sixteen parameters that differed significantly between the two groups (P < 0.05) were used for feature selection by SVM, to find an optimal combination of features for predicting MOF in MSAP and SAP. With the increase in the number of selected features, the acquired combination of features became variable. After feature selection, the combination of nine features, namely HCT, fibrinogen (FIB), D-dimer, reaction time (R-time), kinetic time (K-time), coagulation index (CI), CRP, interleukin-6 (IL-6) and creatinine, obtained the highest AUC value, making it the optimal combination. If other features were added to this basis, the AUC value was reduced (Table 2). The AUC values of the optimal combination, single feature, namely BUN and all features were 0.840 (95% confidence interval (CI): 0.783–0.896), 0.702 (95% CI: 0.625–0.778) and 0.816 (95% CI: 0.755–0.876), respectively (Fig. 1a).

**Table 2:** Different combinations of features by SVM

Combination of features No. of features	HCT	PT	APTT	TT	FIB	D-dimer	R-time	K-time	α	MA	CI	CRP	IL-6	PCT	BUN	Creatinine	AUC
1															√		0.7015
2			√												√		0.7690
3			√										√		√		0.8006
4	√										√		√			√	0.8130
5	√						√	√					√			√	0.8168
6	√				√		√					√	√			√	0.8278
7	√				√					√	√	√	√			√	0.8362
8	√				√		√			√	√	√	√			√	0.8378
9	√				√	√	√	√			√	√	√			√	**0.8396**
10	√				√	√	√	√			√	√	√		√	√	0.8370
11	√				√	√	√	√		√	√	√	√	√		√	0.8382
12	√		√	√	√	√	√	√		√		√	√		√	√	0.8338
13	√	√	√	√	√	√	√	√		√		√	√		√	√	0.8332
14	√		√	√	√	√	√	√		√	√	√	√	√	√	√	0.8301
15	√	√	√	√	√	√	√	√		√	√	√	√	√	√	√	0.8250
16	√	√	√	√	√	√	√	√	√	√	√	√	√	√	√	√	0.8157

Entry in boldface showed highest AUC

### LRA prediction

Similar to SVM, the sixteen features were entered into LRA. However, unlike SVM, the optimal combination of features for predicting MOF only needed six features, including HCT, activated partial thromboplastin time (APTT), FIB, K-time, IL-6 and creatinine. If other features were added to the basis of these six features, AUC would again be reduced (Table 3). The AUC values of the optimal combination, single feature, namely IL-6 and all features were 0.832 (95% CI: 0.773–0.890), 0.709 (95% CI: 0.642–0.775) and 0.783 (95% CI: 0.714–0.853), respectively (Fig. 1b).

**Table 3:** Different combinations of features by LRA

Combination of features No. of features	HCT	PT	APTT	TT	FIB	D-dimer	R-time	K-time	α	MA	CI	CRP	IL-6	PCT	BUN	Creatinine	AUC
1													√				0.7088
2					√											√	0.7803
3					√								√			√	0.8101
4					√			√					√			√	0.8226
5	√				√			√					√			√	0.8294
6	√		√		√			√					√			√	**0.8319**
7	√	√	√		√			√					√			√	0.8275
8	√	√			√			√		√		√	√			√	0.8269
9					√		√	√	√	√	√	√	√			√	0.8285
10		√			√	√	√		√	√	√	√	√			√	0.8240
11			√		√	√	√		√	√	√	√	√		√	√	0.8221
12			√		√	√	√	√	√	√	√	√	√		√	√	0.8147
13		√	√		√	√	√	√	√	√	√	√	√		√	√	0.8149
14	√	√	√		√		√	√	√	√	√	√	√	√	√	√	0.8054
15		√	√	√	√	√	√	√	√	√	√	√	√	√	√	√	0.8040
16	√	√	√	√	√	√	√	√	√	√	√	√	√	√	√	√	0.7833

Entry in boldface showed highest AUC

### ANN prediction

As for SVM and LRA, the same sixteen features were entered into ANN. The optimal combination of features for predicting MOF only required four features, namely HCT, K-time, IL-6 and creatinine. If features were added to the basis of these four features, AUC would be reduced as well (Table 4). The AUC values of the optimal combination, single feature, namely IL-6 and all features were 0.834 (95% CI: 0.777–0.890), 0.705 (95% CI: 0.639–0.772) and 0.789 (95% CI: 0.723–0.856), respectively (Fig. 1c). Thus, HCT, K-time, IL-6 and creatinine were the common important predictive factors for MOF in these three optimal combinations of features obtained by SVM, LRA and ANN.

**Table 4:** Different combinations of features by ANN

Combination of features No. of features	HCT	PT	APTT	TT	FIB	D-dimer	R-time	K-time	α	MA	CI	CRP	IL-6	PCT	BUN	Creatinine	AUC
1													√				0.7054
2													√			√	0.7771
3								√					√			√	0.8077
4	√							√					√			√	**0.8336**
5					√	√					√		√			√	0.8257
6		√			√	√					√		√			√	0.8280
7			√		√	√		√		√			√			√	0.8309
8		√		√	√	√					√	√	√			√	0.8314
9					√	√	√	√	√	√	√		√			√	0.8284
10			√		√	√		√	√		√	√	√		√	√	0.8304
11			√		√	√	√	√		√		√	√	√	√	√	0.8281
12		√	√		√	√		√		√	√	√	√	√	√	√	0.8236
13		√		√	√	√	√	√	√	√		√	√	√	√	√	0.8156
14		√	√	√	√	√	√	√	√	√	√	√	√		√	√	0.8138
15		√	√	√	√	√	√	√	√	√	√	√	√	√	√	√	0.7991
16	√	√	√	√	√	√	√	√	√	√	√	√	√	√	√	√	0.7894

Entry in boldface showed highest AUC

### Comparisons of three models and APACHE II score

We compared the optimal combinations of features resulting from SVM, LRA and ANN and the APACHE II score. The evaluating indexes for these three models and APACHE II score for predicting MOF in MSAP and SAP are shown in Table 5. No significant differences were observed among these three models in SEN, FNR, PPV, NPV and AUC value (*P* > 0.05). The SPE, FPR and predictive accuracy of SVM was superior, but the overall predictive performance of these three models and APACHE II score was not different (P > 0.05) (Fig. 1d).

**Table 5:** Comparison of SVM, LRA, ANN and APACHE II score for predicting MOF

Variable	SVM (95% CI)	LRA (95% CI)	ANN (95% CI)	APACHE II score (95% CI)	P value
SEN	75.00%(63.16–84.13%)	79.17%(67.67–87.50%)	86.11%(75.48–92.78%)	80.56%(69.20–88.59%)	0.413
SPE	81.68%(75.30–86.75%)^b, c^	77.49%(70.78–83.07%)	65.45%(58.19–72.07%)^a^	65.45%(58.19–72.07%)^a^	**< 0.001**
FPR	18.32%(12.84–23.81%)^b, c^	22.51%(16.59–28.43%)	34.55%(27.79–41.31%)^a^	34.55%(27.79–41.31%)^a^	**< 0.001**
FNR	25.00%(15.00–35.00%)	20.83%(11.45–32.21%)	13.89%(5.90–21.88%)	19.44%(10.30–28.58%)	0.413
PPV	60.67%(49.72–70.69%)^c^	57.00%(46.72–66.73%)	48.44%(39.58–57.39%)	46.77%(37.83–55.92%)	0.129
NPV	89.66%(83.91–93.58%)	90.80%(85.01–94.58%)	92.59%(86.45–96.16%)	89.93%(83.38–94.18%)	0.827
Accuracy	79.85%(74.10–83.80%)^b, c^	77.95%(72.94–82.96%)	71.10%(65.62–76.58%)	69.58%(64.02–75.14%)^a^	**0.014**
AUC	0.840 (0.783–0.896)	0.832 (0.773–0.890)	0.834 (0.777–0.890)	0.814 (0.759–0.869)	–

^a^Compared with LRA, P < 0.05

^b^Compared with ANN, P < 0.05

^c^Compared with APACHE II score, P < 0.05

*P* value denoted the overall statistical result for the three models and APACHE II score

Entries in boldface showed significant difference

## Discussion

MOF is a serious systemic complication of AP, leading to high mortality [1]. The 2012 revised Atlanta classification of AP stated that organ failure lasting for more than 48 h is the key determinant of severity [1]. Meanwhile, organ failure is a risk factor for infected pancreatic necrosis and directly increases mortality to 30% [14]. Cardiovascular system failure and respiratory system failure account for 21.1 and 12.3% of total death in AP, and kidney failure and disseminated intravascular coagulation account for 7.0% [15]. Although tryptophan metabolism inhibition is a novel therapeutic blockade for MOF in animal model, no specific therapies are available that are capable of protecting individuals against MOF induced by AP [16]. Therefore, it is extremely important to predict the risk of MOF early. Presently, investigation is still going on for a convenient and practical tool for MOF prediction. Machine-learning techniques have extraordinary information analyzing capabilities and can select the most meaningful features to construct a model; they are novel tools in medical research and have become recognized as such by more and more medical professionals recently [17]. Here, we applied three types of machine-learning algorithms (SVM, LRA and ANN) to the data for AP to develop a convenient tool for predicting the risk of MOF in the medium or late phase of pancreatitis. Clinical data on routine blood test, coagulogram, TEG, inflammatory markers, and renal function were collected and used for machine-learning algorithms. Finally, these three models all yielded satisfactory predictive performance and each produced an optimal combination of features as predictive model.

HCT, K-time, IL-6 and creatinine were common important predictive factors for MOF selected by SVM, LRA and ANN. An elevated HCT is associated with hypovolemia, while decreased HCT suggests hemodilution. It was reported that HCT ≥ 44% could predict persistent organ failure [18], while our research showed that a decreased HCT is correlated with MOF. Therefore, we speculate that a significantly elevated or decreased HCT could indicate a poor prognosis. IL-6 is an effective indicators for the degree of inflammatory response in AP [19]. Plasma IL-6 levels are markedly increased in pancreatitis animals [20], and inhibition of IL-6 alleviates the formation of edema, inflammatory cell infiltration, and necrosis in cerulein-induced AP [21]. IL-6 is a pro-inflammatory cytokine and regulates leukocyte recruitment through the IL-6 trans-signaling-dependent STAT3 pathway in pancreatic acinar cells. It links local inflammation in the pancreas to systemic inflammation, and even to lethal extrapancreatic organ damage [22]. IL-6 levels are significantly higher in patients with acute lung injury compared with MAP patients [22]. Our results show that patients with MOF had higher levels of IL-6 than those without MOF and demonstrate that IL-6 plays an important role in predicting the risk of MOF.

AP patients with organ failure were reported to have higher prothrombin time (PT) and APTT levels than those without organ failure, but PT and APTT were not able to independently predict organ failure in a multivariate analysis [23]. Here, patients with MOF had higher PT, APTT, thrombin time (TT), and D-dimer and lower FIB levels, suggesting that the coagulation dysfunction was more serious in patients with MOF. However, none of these coagulogram parameters were entered into the three predicted models for MOF in pancreatitis simultaneously.

K-time, a parameter in TEG which is a comprehensive examination reflecting coagulation state, like alpha (α) angle mainly indicates FIB level, but also can be influenced by platelet function to a small extent. Other parameters in TEG included R-time, which indicates the role of clotting factors, maximum amplitude (MA), which indicates the number and function of platelet, and CI indicating the overall coagulation status [24]. Prolonged R-time and K-time suggest a state of hypocoagulation in pancreatitis patients with MOF, while α angle, MA and CI would be reduced. Here, K-time was demonstrated to be vital in these three models. The reasons that K-time can predict MOF are the following: firstly, one of the consequences of local inflammation is vascular injury within the pancreas, leading to endothelial cell activation and damage, increased vascular permeability, leukocyte adhesion and migration, and activation of the coagulation system [25]. Secondly, some clotting factors concentrations alter due to activation of the coagulation system; for example, concentrations of serum tissue factor and von Willebrand factor increase in SAP [26, 27], and this could significantly predict acute lung injury [27]. This results in the hypercoagulable state and thrombotic complications including thrombosis and gangrene observed in some SAP patients [28]. Then, the consumption of large amounts of clotting factors leads to a hypocoagulable state. In turn, coagulation dysfunction aggravates inflammation because thrombin promotes the production and release of pro-inflammatory cytokines, particularly IL-6 [28]. Therefore, coagulation dysfunction reflects the severity of AP, and the positive feedback relationship between coagulation and inflammation is the reason that K-time could predict MOF in MSAP and SAP.

The serum creatinine level helps to predict organ failure in SAP if it is higher than 110 μmol/L [29]. Our results showed levels of creatinine in patients with MOF were higher than in patients without MOF. Therefore, creatinine, an essential indicator of renal function, is capable of predicting the risk of MOF in the medium or late phase of pancreatitis.

AP patients with hyperlipidemia had a higher mortality rate, worse prognosis and higher risk of local complications [30], because elevated level of triglyceride and free fatty acids lead to toxic effects and are essential risk factors for pancreatic acinar cell damage [31]. Diabetes and hypertension have been reported to increase the risk of AP [32] and could be predictors of SAP [33]. Age and BMI are recognized factors to assess the severity of AP initially [10]. However, AP’s etiology, such as hyperlipidemia, diabetes or hypertension, age and BMI, were not capable of predicting MOF in MSAP and SAP in this study, probably because these parameters are less related to MOF than inflammatory and coagulation parameters.

Here, we made use of machine learning to predict the risk of MOF induced by pancreatitis. In one study, ANN was able to predict the incidence of portosplenomesenteric venous thrombosis in AP, with an AUC value of 0.849. However, that was a small-sample research analyzing only 11 parameters [34]. We conducted a two-step feature selection strategy to develop a superior prediction model. The first step eliminated a great number of unrelated data. Then, five-fold cross-validation was used to test the predictive ability of the three models investigated to achieve a reliable and stable predictive model.

As for our study, it is very convenient to get the predicted probability for MOF of an individual, which is superior to complicated score systems such as APACHE II score. Secondly, compared to traditional statistical methods, SVM, LRA and ANN are better at analyzing nonlinear relationships between various biochemical markers and MOF. In addition, these three models are practical, since the parameters used in the three models are well established in routine clinical work. We recommend the ANN model, which only needs four parameters to get satisfactory AUC values, as well as SVM, LRA and the APACHE II score. Moreover, we note that combining coagulation and inflammation parameters has great potential for predicting the risk of MOF, confirming the effect of coagulation dysfunction in the pathogenesis of MOF induced by AP.

## Conclusions

Three convenient and practical models which can predict the risk of MOF of individual AP patients based on SVM, LRA and ANN were developed and validated. HCT, K-time, IL-6 and creatinine play a significant role in these models. All of the parameters in the three models are well established in routine clinical work, so SVM, LRA and ANN could be promising tools for predicting MOF in MSAP and SAP patients in the clinical practice.

## Additional files

Additional file 1: **Table S1.** Laboratory data obtained on admission of all patients. (DOC 36 kb)

Additional file 2: **Table S2.** Baseline characteristics in the whole cohort of patients. APACHE II score, Acute Physiology and Chronic Health Evaluation II score; BMI, body mass index; MOF, multiple organ failure. (DOC 32 kb)

## Data Availability

The datasets during and/or analysed during the current study are available from the corresponding author on reasonable request.

## Abbreviations

ANN: Artificial neural networks
APACHE II score: Acute Physiology and Chronic Health Evaluation II score
APTT: Activated partial thromboplastin time
AUC: Area under the curve
BMI: Body mass index
BUN: Blood urea nitrogen
Ca^2+^: Calcium ion
CI: Coagulation index
CI: Confidence interval
CRP: C-reactive protein
FIB: Fibrinogen
FNR: False negative rate
FPR: False positive rate
HCT: Hematocrit
IL-6: Interleukin-6
K-time: Kinetic time
LRA: Logistic regression analysis
LY30: Percent fibrinolysis at 30 min
MA: Maximum amplitude
MOF: Multiple organ failure
MPV: Mean platelet volume
NEUT: Percentage of neutrophils
NPV: Negative predictive value
PCT: Procalcitonin
PDW: Platelet distribution width
PLT: Platelet count
PPV: Positive predictive value
PT: Prothrombin time
R-time: Reaction time
SEN: Sensitivity
SPE: Specificity
SVM: Support vector machine
TEG: Thrombelastogram
TT: Thrombin time
WBC: White blood cell
α angle: Alpha angle
