# Supplementary material for: Development and validation of three machine-learning models for predicting multiple organ failure in moderately severe and severe acute pancreatitis
Source: BMC Gastroenterol. 2019 Jul 4;19:118. doi: 10.1186/s12876-019-1016-y (PMC6611034; doi:10.1186/s12876-019-1016-y)
Supplement: Supplementary file 1 — Table S1. Laboratory data obtained on admission of all patients. (DOC 36 kb) [file 12876_2019_1016_MOESM1_ESM.doc]

Supplementary Table S1. Laboratory data obtained on admission of all patients

| Parameters | | |
| --- | --- | --- |
| Routine blood test | | |
| White blood cell (WBC) | Percentage of neutrophils (NEUT) | Hematocrit (HCT) |
| Platelet count (PLT) | Mean platelet volume (MPV) | Platelet distribution width (PDW) |
| Coagulogram | | |
| Prothrombin time (PT) | Activated partial thromboplastin time (APTT) | Thrombin time (TT) |
| Fibrinogen (FIB) | D-dimer |  |
| Thrombelastogram (TEG) | | |
| Reaction time (R-time) | Kinetic time (K-time) | Alpha angle (α angle) |
| Percent fibrinolysis at 30 min (LY30) | Maximum amplitude (MA) | Coagulation index (CI) |
| Inflammatory markers |  |  |
| C-reactive protein (CRP) | Interleukin-6 (IL-6) | Procalcitonin (PCT) |
| Renal function | | |
| Blood urea nitrogen (BUN) | Creatinine | Calcium ion (Ca^2+^) |
