# Supplementary material for: Development and validation of three machine-learning models for predicting multiple organ failure in moderately severe and severe acute pancreatitis
Source: BMC Gastroenterol. 2019 Jul 4;19:118. doi: 10.1186/s12876-019-1016-y (PMC6611034; doi:10.1186/s12876-019-1016-y)
Supplement: Supplementary file 2 — Table S2. Baseline characteristics in the whole cohort of patients. APACHE II score, Acute Physiology and Chronic Health Evaluation II score; BMI, body mass index; MOF, multiple organ failure. (DOC 32 kb) [file 12876_2019_1016_MOESM2_ESM.doc]

Supplementary Table S2. Baseline characteristics in the whole cohort of patients

|  | Patients, (n =263) |
| --- | --- |
| Male, no. (%) | 165(62.73) |
| Median age, year | 47(39,59) |
| History of hypertension, no. (%) | 58(22.05) |
| History of diabetes, no. (%) | 31(11.79) |
| Etiology, no. (%)  Biliary  Hypertriglyceridemia  Alcoholic  Other | 106(40.30)  92(34.98)  24(9.13)  41(15.59) |
| BMI, kg/m2  Obese (BMI≥25kg/m2), no. (%) | 25.71(23.53,27.92)  147(55.89) |
| MOF, no. (%) | 72 (27.38) |
| APACHE II score | 10(8,13) |
